# Phosphorylation of EZH2 differs HER2-positive breast cancer invasiveness in a site-specific manner

**DOI:** 10.1186/s12885-023-11450-9

**Published:** 2023-10-06

**Authors:** Feng Yu, Lili Li, Mengwen Zhang, Shanshan Sun

**Affiliations:** 1 Department of Colorectal Surgery and Oncology, Key Laboratory of Cancer Prevention and Intervention, The Second Affiliated Hospital, Ministry of Education, Zhejiang University School of Medicine, Hangzhou, Zhejiang, China; Cancer Institute, Zhejiang University, Hangzhou, 310058 Zhejiang China; 2 Department of Medical Oncology, Second Affiliated Hospital, Zhejiang University School of Medicine, Hangzhou, 310058 China; 3 Department of Plastic Surgery, Second Affiliated Hospital, Zhejiang University School of Medicine, Hangzhou, 310058 China; 4 Department of Breast Surgery, Second Affiliated Hospital, Zhejiang University School of Medicine, Hangzhou 310058, China; Key Laboratory of Tumor Microenvironment and Immune Therapy of Zhejiang Province, Second Affiliated Hospital, Zhejiang University School of Medicine, Hangzhou, 310058 China

**Keywords:** EZH2, Phosphorylation, HER2-Positive BC, Invasiveness, Metastasis

## Abstract

HER2-positive breast cancer (BC) invasiveness and drug-resistance issue is the critical treatment obstacle recently. We investigated the total and phosphorylated status EZH2 expression in database and BC tissue microarray. We demonstrated for the first time that EZH2 is distributed both in cytoplasm and nucleus of breast cancer cells in a phosphorylation site-specific manner. High expressed-EZH2 cases more frequently had an advanced clinical stage (lymph node metastasis) and aggressive features than EZH2-low cases, potentially indicating the high risk of HER2-positive BC (p < 0.05). Notably, highly expressed phosphorylated EZH2 is differently located in cytoplasm or nucleus in a site-specific manner in breast cancer cells. Nucleus-located pEZH2-S21 is expressed in invasive and lymph node metastatic HER2-positive BC cases (p = 0.144, p = 0.001). Cytoplasmic pEZH2-T487 is correlated with HER2 positive status (p = 0.014).

In conclusion, high expression of nucleus-located EZH2 might be a predictor of invasive BC. Activation of phosphorylated EZH2-S21 site in nucleus would be a potential predictor of HER2-positve BC and poor efficacy of HER2-target therapy. These results point to a PRC2-independent non-epigenetic mechanism and therapeutic strategy of EZH2 in HER2-positive BC.

**Supplementary Information:**

The online version contains supplementary material available at 10.1186/s12885-023-11450-9.

## Introduction

Human epidermal growth factor receptor 2 (HER2)-positive breast cancer (BC) accounts for approximately 15–20% of all types of BC patients. Over the years, HER2-positive BC is associated with an increased risk of recurrence and metastasis and poor outcome because of its more aggressive biological behaviors [1]. Although there are emerging dramatic improvements of anti-HER2 target treatment in the recent years, HER2-positive BC invasiveness and drug-resistance still remain to be a tricky potential molecular issue in current era [2]. Therefore, it is an unmet need for seeking for the precise molecular mechanism of HER2-positive BC aggressive biological behavior.

Enhancer of Zeste Homolog 2(EZH2) is a histone methyltransferase that catalyzes the histone H3 lysine 27 methylation. It is one of the core subunits of the polycomb-repressive complex 2(PRC2), which is consisted of several key components—EED, SUZ12 and RpAp46/48 [3, 4]. Numerous studies have shown that EZH2 is involved in a wide range of biological processes, such as tumor development, growth [5], metastasis [6, 7], apoptosis [8], angiogenesis [9], stem cell renewal/ maintenance [10], and treatment-resistance [11–13]. Indeed, most of these functional integrities of EZH2 are based on epigenetic mechanism. Therefore, EZH2 is considered as a transcriptional silencer in tumors, which has been well established. Nevertheless, minority is focused on the non-epigenetic role in breast cancer, especially the posttranscriptional modification (PTM)---phosphorylation. In comparison to an epigenetic repressor of tumor-related genes in nucleus, little is known about the function of cytoplasmic EZH2 in tumor cells, breast cancer cells particularly.

Apart from epigenetic mechanism and methylation, phosphorylation of EZH2 function in breast tumors has been increasingly taken consideration and revealed in recent years [14]. Accordingly, the subcellular location of EZH2 in tumor cell has also been concerned by researchers. For instance, cytoplasmic pEZH2 T367 expression and subcellular localization could stratify metaplastic carcinoma subtypes [15]. In addition, pEZH2-T367 induces EZH2 cytoplasmic localization to promote breast cancer metastasis [16]. Similarly, it is found that high cytoplasmic EZH2 expression is significantly associated with short overall survival in cholangiocarcinoma [17]. Evidence shows that EZH2 in the cytoplasm is closely coupled to cancer stem cell properties [18], thus inhibition of cytoplasmic EZH2 induces antitumor activity in NSCLC [19]. As aforementioned, the extranuclear function of EZH2 in tumorigenesis has been gradually noticed and validated in solid tumors, less has been explored in HER2-positive BC concerning EZH2, especially in aggressiveness and drug-resistance.

Therefore, we examined the non-traditional biological function of phosphorylated EZH2 in breast cancer. In this work, we take the lead to reveal a unique and non-classical role of EZH2 phosphorylation in HER2-positive BC, particularly in a site-specific manner with different cell components.

## Results

### EZH2 is highly expressed in malignancies and correlated with poor prognosis of breast cancer

**Fig. 1:**
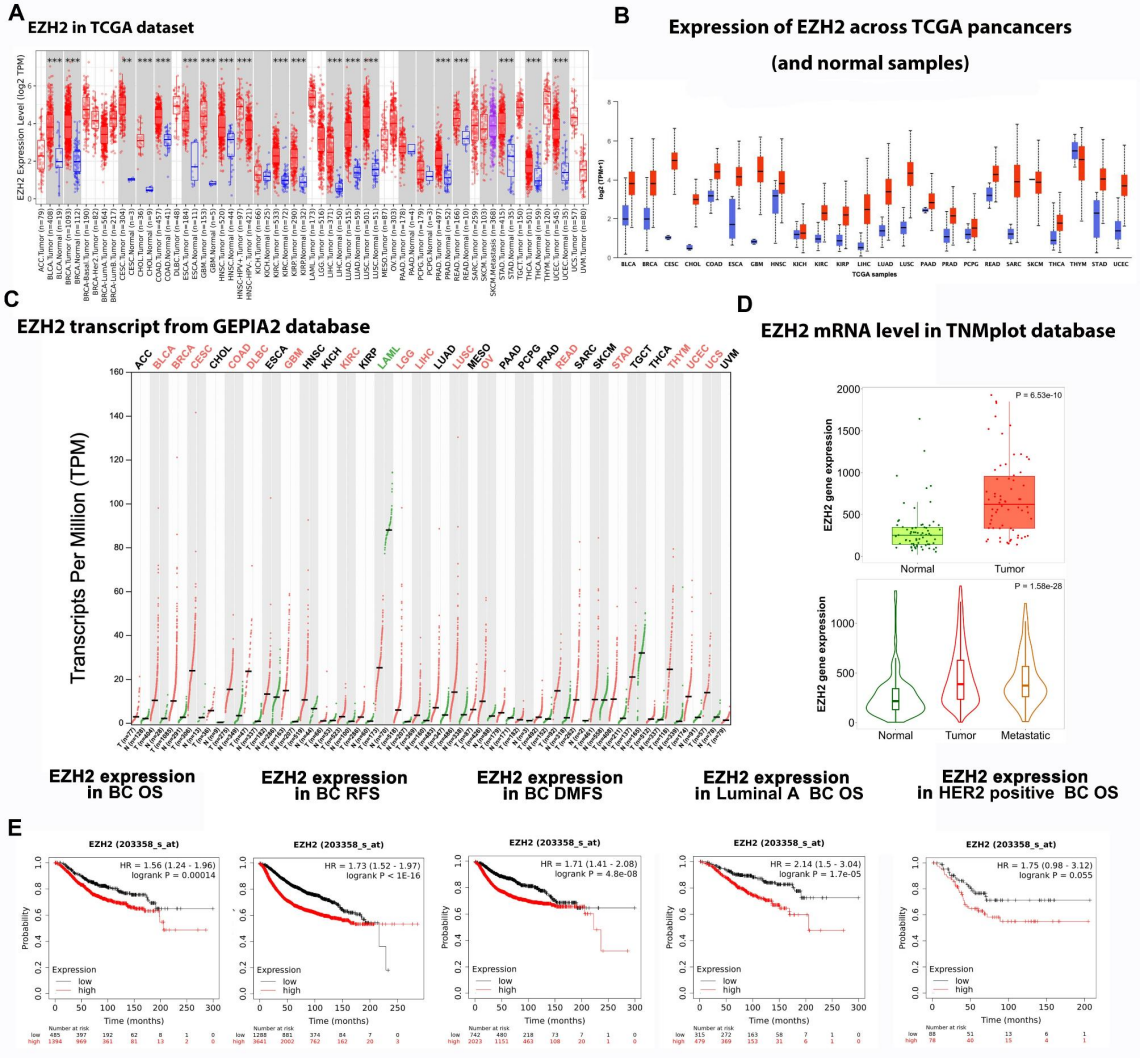
EZH2 expression in tumor and normal tissues and its prognosis of BC in database. **A.B** EZH2 expression level in pancancers and normal samples in TCGA dataset. **C**. EZH2 transcript in pancancers in GEPIA2 database. **D**. EZH2 mRNA level in TNMplot database. **E**. BC patients’prognosis of EZH2 expression in K-M plotter

We explore the expression of EZH2 gene in pan-cancer tissues as well as normal tissues in The Cancer Genome Atlas (TCGA) RNAseq data set (TCGA). Data shows that EZH2 expression mRNA level in pan-cancer including breast cancer tissue is higher than the corresponding normal tissue in TCGA dataset (Fig. 1A-B). Consistently, the EZH2 gene transcript from Gene expression profiling interactive analysis (GEPIA2) dataset and TNMplot database also provide that higher level in breast cancer and metastasis specimens of EZH2 than normal mammary glands epithelium specimens (Fig. 1C-D). To explore the impact of EZH2 in breast cancer on BC patients’ survival, we analysis the correlation between EZH2 mRNA level and survival prognosis in different subtypes of BC patients in K-M plotter database (http://kmplot.com/analysis). Data shows that Patients with lower EZH2 expression had longer overall survival (OS), recurrence-free survival (RFS) and distant metastasis-free survival (DMFS) in total breast cancer dataset (HR = 1.56, p < 0.05; HR = 1.73, p < 0.05; HR = 1.71, p < 0.05). Of note, patients with higher EZH2 expression level had poor OS for luminal A and HER2-positive BCs (HR = 2.14, p < 0.05; HR = 1.75, p = 0.055) (Fig. 1E).

### EZH2 exerts cytoplasm-nucleus sublocation in breast cancer tissues, which associated with invasiveness

Two tissue microarray (TMA) slides of breast cancer specimen totally containing 16 benign tissues, 7 DCIS tissues and 90 breast cancer tissues (113 cases in total) were collected and produced by our department and lab from the Second Affiliated Hospital, Zhejiang University School of Medicine, Hangzhou. The total specimen samples were classified as normal mammary and benign lesion, Ductal carcinoma in situ (DCIS), Invasive ductal carcinoma (IDC) and other invasive type breast carcinoma for further investigation. Besides, we stratified breast tumors into luminal-type, HER2-positive and Triple negative breast cancer (TNBC) subtypes for the following study.

**Fig. 2:**
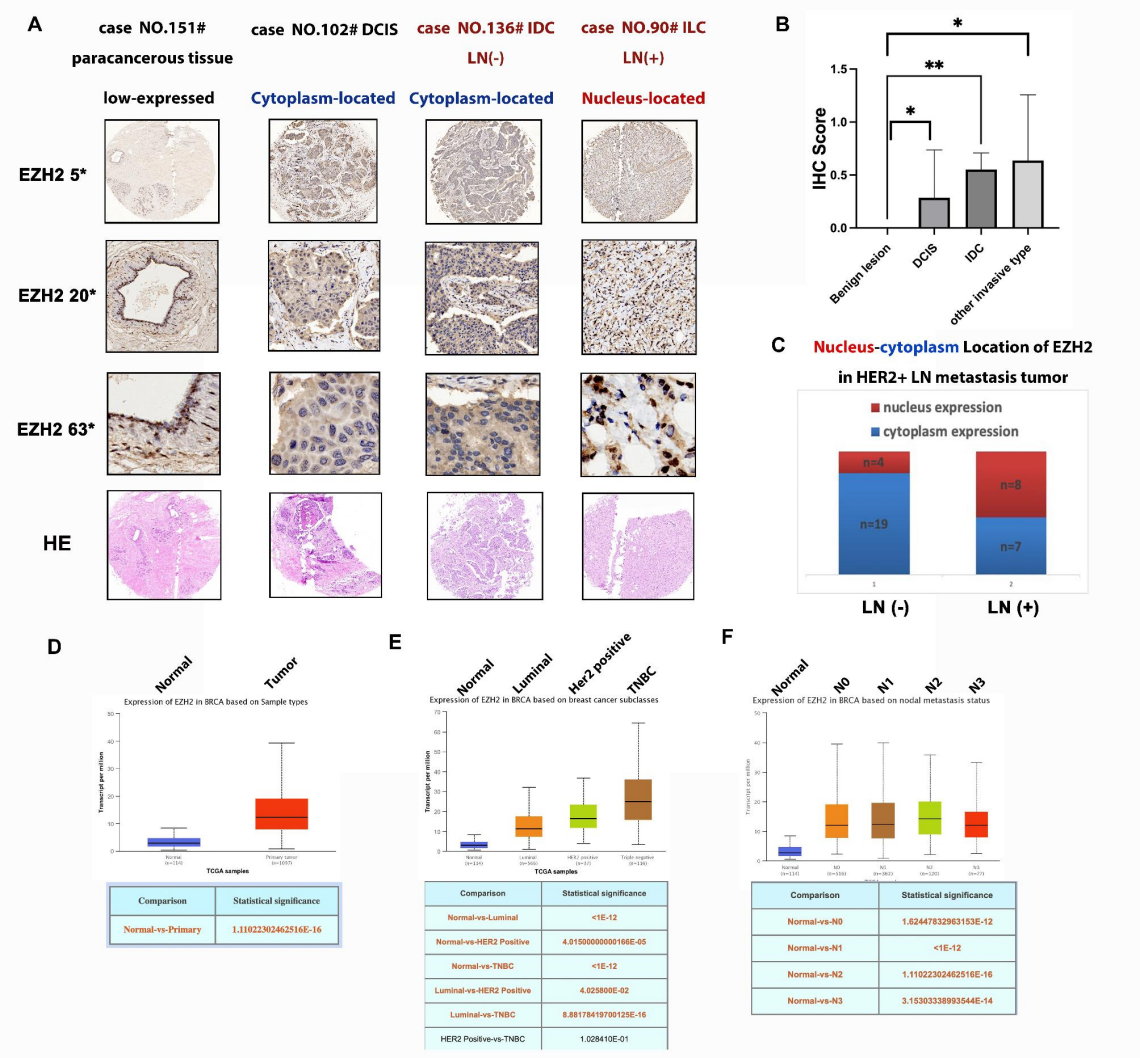
EZH2 expression in BC tissues and its clinical significance of BC patients. (**A**) Representative IHC staining of EZH2 expression in paracancerous breast tissue, DCIS, lymph node negative-IDC and lymph node metastasis IDC tissue as low and high expression with different sublocations. (The immunohistochemical images were at 5,20,63x magnification) (**B**) EZH2 expression IHC score in paracancerous breast tissue, DCIS and other invasive type tissues by statistical analysis. (**C**) Nucleus-cytoplasm location of EZH2 in LN metastasis BC tissue. **D**,**E**,**F**. EZH2 expression in BC tissue, different subclasses and from TCGA database

It could be observed that EZH2 was highly expressed in both cytoplasm and nucleus of breast cancer tissues. In benign lesion, EZH2 staining is almost negative or weak in IHC assay. In DCIS tissues, 7 cases were almost positive for EZH2 expression as cytoplasm location (Fig. 2A, B). Strikingly, we found that the sublocation of EZH2 staining was associated with lymph node metastasis in HER2-positive invasive BC tissues—82.6% (19/23) cases were positive for EZH2 expression in LN negative IDC specimens, as cytoplasm-located staining while 53.3% (8/15) cases were positive for EZH2 expression in LN positive IDC specimens as nucleus-located staining (Fig. 2C, p < 0.05). In TCGA database, Fig. 2D show that EZH2 expression in breast cancer is significantly higher than normal breast tissue. Simultaneously, EZH2 expression level is statistically higher in aggressive subtypes of BC—TNBC and HER2-positive subtype (p < 0.05, Fig. 2E). To determine EZH2 expression in metastatic breast cancer, we make comparison of EZH2 in each breast cancer N stage group (N0:516 cases, N1:362cases, N2:120cases, N3:77cases) with normal mammary gland tissue (n = 114 cases) based on TCGA database. Consistently, data shows that EZH2 expression level is statistical significantly higher in high N stage of BC than normal breast epithelium group (p < 0.05, Fig. 2F). Consequently, the cytoplasm-nucleus sublocation of EZH2 in BC tissues is closely related to BC invasiveness, HER2-positive BC, in particular.

### Phosphorylation of EZH2 T487 and S21 site display obvious importance in HER2-positive BCs

**Fig. 3:**
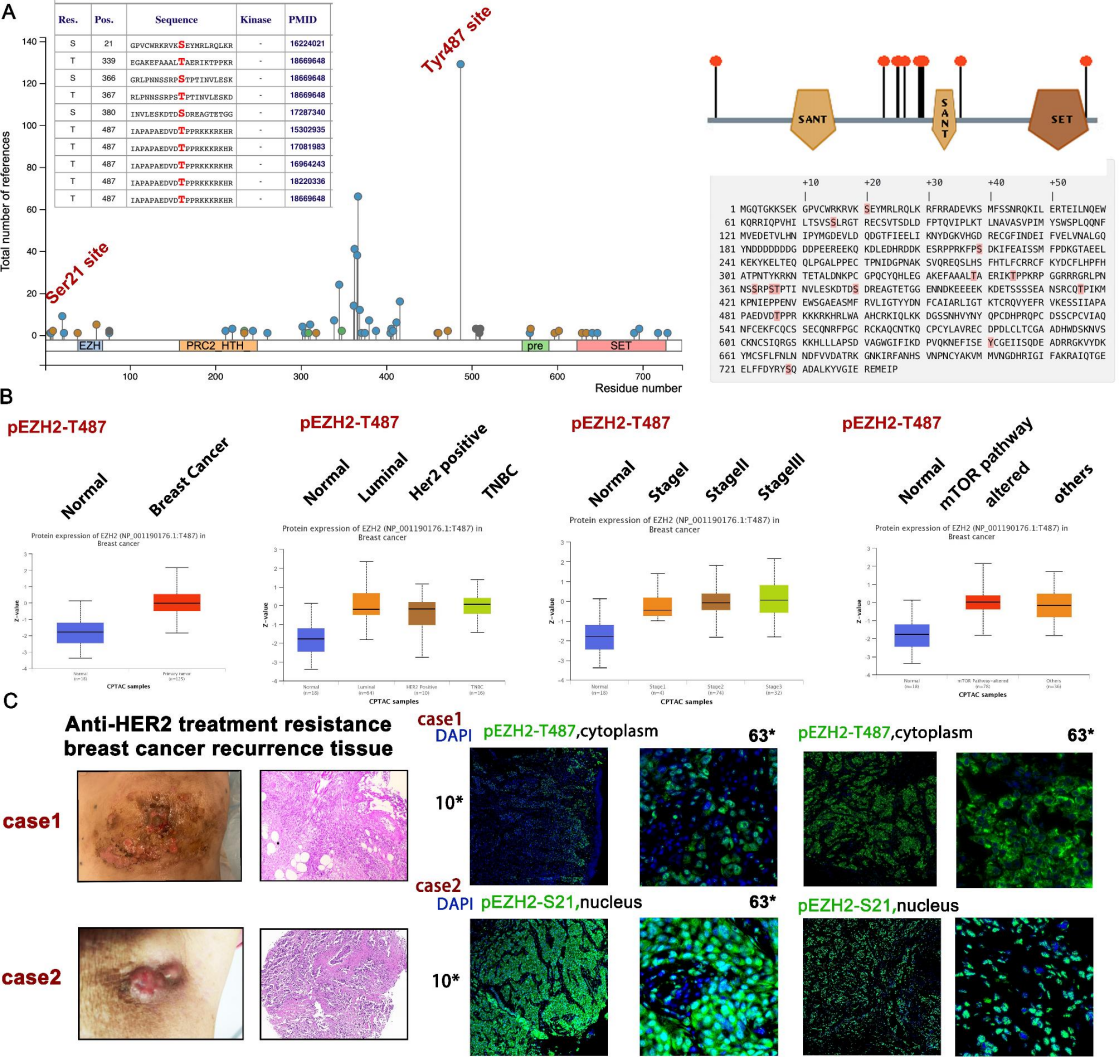
Phosphorylated EZH2 (S21 and T487) profile in breast cancer tissues in database and clinical specimens. (**A**) All phosphorylated sites and sequences of EZH2 are displayed in schematic diagram. (**B**) Phosphorylated EZH2-T487 level in normal and tumor tissue, different subtypes of BC, different tumor stages and mTOR pathway alteration in CPTAC dataset. (**C**) phosphorylated EZH2 (S21 and T487) in anti-HER2 treatment resistance BC recurrence tissue

All phosphorylated sites of EZH2 are displayed in schematic diagram of Fig. 3A from PhosphoSitePlus database. The sequence of each S, T, Y amino acid phosphoSite of EZH2 is listed from PhosphoELM database (http://phospho.elm.eu.org). We analysed pEZH2-T487 site of breast cancer in Clinical Proteomic Tumor Analysis Consortium (CPTAC) database. It is found that pEZH2-T487 expression level is increased in breast cancer specimen (n = 125) than normal tissues (n = 18), especially in luminal (n = 64) and TNBC (n = 16) subtype (Fig. 3B). Moreover, pEZH2-T487 is positively associated with late BC tumor stage—pEZH2-T487 level is gradually elevated in stage I, II and III in CPTAC BC samples than normal tissues (Fig. 3B). In addition, it suggested that higher level of pEZH2-T487 is presented in mTOR altered BC tissues, which might imply underlying signaling pathway mechanisms uncovered (Fig. 3B).

**Fig. 4:**
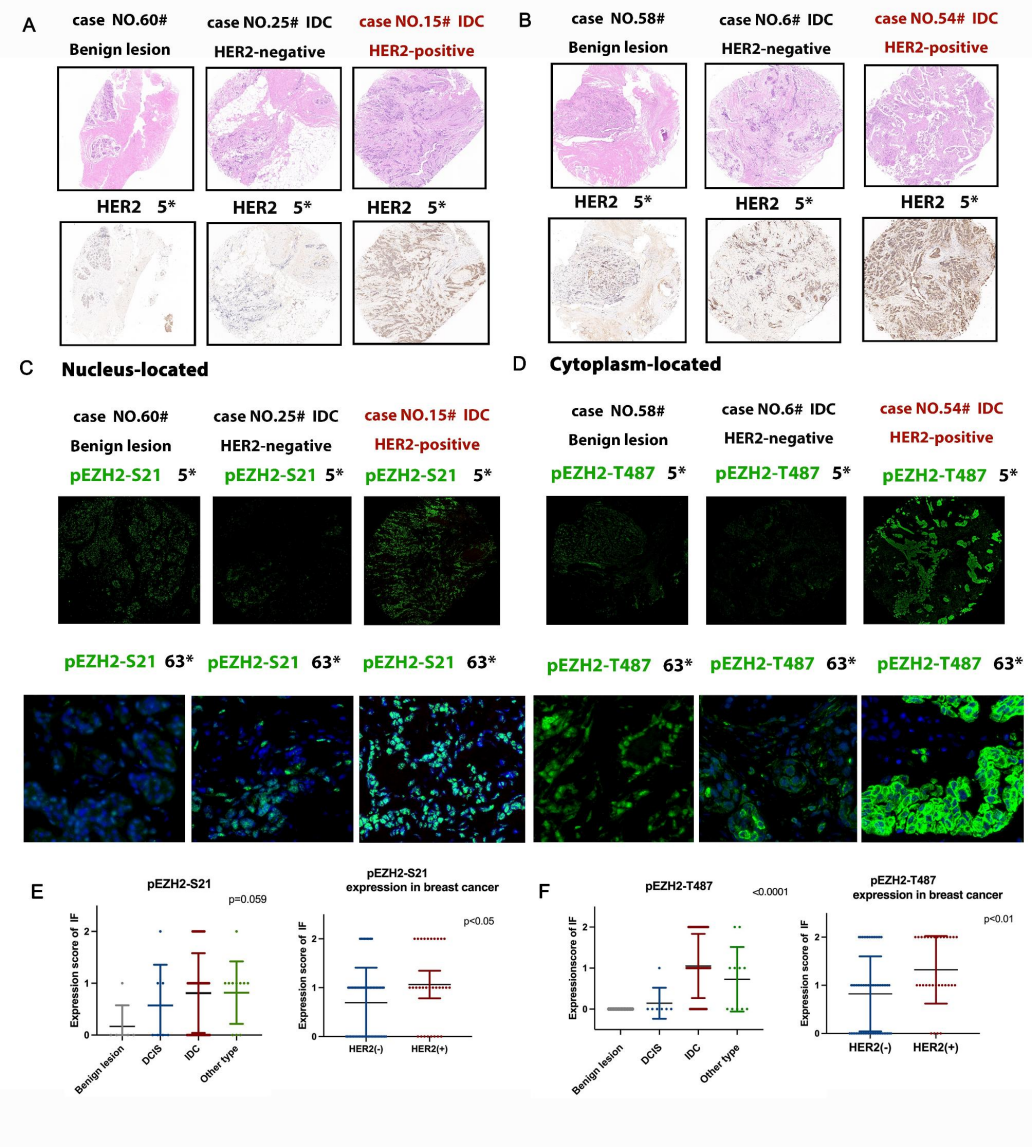
Phosphorylation of EZH2 (Serine21, Tyrosine487 site) correlates with invasive and HER2-positive BC in different subcellular components. **A**.**B** Representative HE and HER2 IHC staining images from human breast benign lesion, HER2-negative IDC and HER2-positive IDC. **C**. Representative pEZH2-S21 IF staining images from human breast benign lesion, HER2-negative IDC and HER2-positive IDC. **D**. Representative pEZH2-T487 IF staining images from human breast benign lesion, HER2-negative IDC and HER2-positive IDC. **E**.**F** Expression level of pEZH2-S21 and pEZH2-T487 in breast benign lesion, DCIS, IDC and other type tissue by statistical analysis. Expression level of pEZH2-S21 in HER2-negative and HER2-positive BC tissue by statistical analysis

Early in 2018, the author and her colleges have revealed that transcription factor STAT3 enhances lincRNA HOTAIR transcription by interacting with pEZH2-Serine21, instead of pEZH2-T487 in head and neck squamous cell carcinoma (HNSCC), thus promoting tumor progression and cisplatin resistance [20]. To validate the phosphorylation status of EZH2 in invasive metastatic BC, we examined pEZH2-T487 and pEZH2-S21 expression by immunofluorescence two individual HER2-positive BC chest recurrence case tissue after multi-line anti-HER2 treatment. With one accord, images show that pEZH2-T487 is almost intensively staining in cytoplasm while pEZH2-S21 is almost intensively staining in nucleus in the anti-HER2 treatment recurrence BC tissues (Fig. 3C). Subsequently, we examined pEZH2-S21, pEZH2-T487 and HER2 staining in BC specimens by IF and IHC assay. The clinicopathological characteristics of BC patients in TMA were collected and analyzed statistically using Pearson’s chi-square test. In Table 1, data shows that cytoplasmic pEZH2-T487 is correlated with HER2 positive status of breast cancer tissues (p = 0.014). Meanwhile, nucleus-located pEZH2-S21 is expressed in invasive and lymph node metastatic HER2-positive BCs (p = 0.001, Table 2). Representative HE and HER2 Immunohistochemical images of benign lesion of breast (case No.60, No.58 on TMA), HER2-negative IDC (case No.25, No.6 on TMA) and HER2-positive IDC (case No.15, No.54 IDC on TMA) are displayed in Fig. 4A and B, respectively. Consistently, it is suggested that both pEZH2-S21(p = 0.059) and pEZH2-T487 (p < 0.0001) expression level are increased in IDC than benign lesion, DCIS and other invasive type tissue (Fig. 4E, F). Remarkably, pEZH2-S21(p < 0.05) and pEZH2-T487 (p < 0.01) are strictly correlated with HER2 status, respectively (Fig. 4E, F). Accordingly, we observed an increased EZH2 phosphorylation levels of T487 and S21 site in breast cancer tissues. Interestingly, the T487 site of phosEZH2 and S21 site of phosEZH2 are localized differently in subcellular segment of tumor tissues—cytoplasm and nucleus, respectively (Fig. 4C, D). The intensity of pEZH2-T487 is significantly stronger in HER2-positive IDC as cytoplasmic staining than in HER2-negative IDC, as well as in normal breast tissue (Fig. 4D). Meanwhile, the intensity of pEZH2-S21 is significantly stronger in HER2-positive IDC as nuclear staining than in HER2-negative IDC, as well as in normal breast tissue (Fig. 4C). This phenomenon of phosphorylated EZH2 cytoplasm-nucleus sublocation difference in site-specific manner is of statistical significance in our TMA data (p < 0.05).

**Table 1:** pEZH2-T487 in breast cancer tissue

Characteristics	No. of patients (%)				P
	Total(n = 113)	Negative (n = 49)	Moderate (n = 36)	Intensive (n = 28)	
**Histopathological**					**< 0.001**
Normal, benign lesion	16	16	0	0	
DCIS	7	6	1	0	
IDC	79	22	31	26	
Other invasive type	11	5	4	2	
**PR status**					0.236
Negative	46	12	17	17	
Positive	49	19	19	11	
**HER2 status**					**0.014**
Positive	31	4	13	14	
Negative	62	25	23	14	
**Ki67**					0.544
high	23	7	7	9	
low	68	21	28	19	
**LN status**					0.718
LN-	56	21	19	16	
LN+	36	11	15	10	
**T**					**0.107**
< 2 cm	39	14	14	11	
2-4 cm	37	8	15	14	
> 4 cm	19	11	5	3	
**WHO grade**					0.692
1	1	0	1	0	
2	53	17	19	17	
3	79	6	11	8	
**pEZH2-S21 expression**					**< 0.001**
Negative	54	44	8	2	
Moderate	40	4	23	13	
Intensive	19	1	5	13	
**Total EZH2 expression**					**< 0.001**
Negative	72	42	17	13	
Moderate	28	4	10	14	
Intensive	12	3	8	1	

**Table 2:** pEZH2-S21 in breast cancer tissue

Characteristics	No. of patients (%)				P
	Total (n = 103)	Negative (n = 44)	Moderate (n = 40)	Intensive (n = 19)	
**Histopathological**					0.226
Normal, benign lesion	6	5	1	0	
DCIS	7	4	2	1	
IDC	79	32	30	17	
Other invasive type	11	3	7	1	
**PR status**					0.719
Negative	46	16	20	10	
Positive	49	21	19	9	
**HER2 status**					**0.001**
Positive	31	8	13	10	
Negative	62	28	25	9	
**Ki67**					0.198
high	23	6	10	7	
low	68	30	27	11	
**LN status**					**0.144**
LN-	56	23	20	13	
LN+	36	15	18	3	
**T**					0.409
< 2 cm	39	19	13	7	
2-4 cm	37	11	17	9	
> 4 cm	19	9	8	2	
**WHO grade**					0.841
1	1	0	1	0	
2	53	20	23	10	
3	25	10	10	5	
**pEZH2-T487 expression**					**< 0.001**
Negative	49	44	4	1	
Moderate	36	8	23	5	
Intensive	28	2	13	13	
**Total EZH2 expression**					**< 0.001**
Negative	72	46	18	8	
Moderate	28	4	14	10	
Intensive	12	4	7	1	

Regarding to the target genes of EZH2 in breast cancer, we performed Bioinformatics analysis based on TCGA-BRCA using STRING, and metascape (http://metascape.org) database, which illustrated in Supplementary Fig. 1. Genes such as DBF4 (R = 0.72, p < 0.01), CDCA8 (R = 0.75, p < 0.01), RAD54L (R = 0.71, p < 0.01) and STMN1 (R = 0.79, p < 0.01) are correlated with EZH2 expression, which have been displayed in sup. Figure 1 C. Ezh2 has also involved in several signaling pathways, such as mitosis cell cycle, DNA-damage and cellular response, etc. using Enriched Ontology Clusters (sup. Figure 1D, E) .

More specifically, we employed a highly aggressive breast cancer cell line (BT549) treated with EZH2 inhibitor (Dznep) for RNA sequencing assays, attempting to identify its downstream target genes in BC in vitro. Data was analyzed by Gene ontology (GO) enrichment and Kyoto Ency- clopedia of Genes and Genomes (KEGG) pathway. GO analysis for differentially expressed genes in EZH2 inhibition group and control group. Differentially expressed genes (DEGs) driven by EZH2 is obtained as gene correlation network figure exhibited as “pathway in cancer” and “metabolic pathway” panel, as representatives (Supplementary Fig. 2).

The downstream target genes of EZH2 would provide molecular clues for our further investigation.

Taken together, our findings demonstrate for the first time that the expression and subcellular localization of phosphorylated EZH2 differs HER2-positive breast cancer invasiveness in a site-specific manner, suggesting a correlation with lymph node metastasis. Therefore, further clinical diagnostic and treatment implications would be provided for HER2-positive BCs.

## Discussion

The methyltransferase EZH2 play vital role in chromatin conformation regulation and gene epigenetic transcription [21]. EZH2 has been implicated to have critical role in multiple carcinogenesis and tumor development and progression, including breast cancer [22]. Although various studies have been investigated in EZH2 behavior in malignancies, few literatures have been focused on the cytoplasm-nucleus sublocation of EZH2 in tumor cells. Interestingly, we initially report and uncover a novel evidence that phosphorylated EZH2 behaves as an actionable target in HER2-positive BC in a site-specific manner. In this study, we present significant evidence to reveal that phosphorylated EZH2 is differently located in cytoplasm and nucleus of breast cancer cells in tumor tissue specimen in a site-specific manner, which suggesting pEZH2 is critical for identify invasiveness and metastasis in HER2-positive BC.

The biological function of phosphorylated EZH2 in tumor cells have been increasingly raising concern recently. Data shows that it is multifaceted and context-dependent. For instance, phosphorylation of EZH2 at T311 inhibits PRC2 methyltransferase activity to suppress tumorigenesis [23]. In natural killer/T-cell lymphoma, EZH2 S220 phosphorylation is increased by MELK to mediate its sensitivity to bortezomib [24]. CDK2-mediated phosphorylation of EZH2 at T416 activates EZH2 to silence target genes—ERα gene (ESR1), leading to in high-grade serous ovarian carcinoma (HGSOC) and TNBC [25]. Meanwhile, CDK2-mediated EZH2 phosphorylation(pT416) drives tumorigenesis— converts the luminal breast cancer to TNBC [26]. EZH2 phosphorylation at T372 reduces ovarian cancer cell proliferation, migration and tumor formation [27]. Calcium/Calmodulin dependent protein kinase II alpha (CAMK2A), a key calcium signaling molecule, phosphorylates EZH2 at T487 with suppression of EZH2 activity to support Tumor initiating cells (TIC) maintenance in lung adenocarcinoma [28]. Phosphorylation of EZH2 at T435 and T487 by CDK1 and CDK2 epigenetically silences target genes in breast cancers [29, 30]. Mechanically, phosphorylation of Thr-487 site, which mediated by CDK1 is necessary for EZH2 ubiquitination and subsequent degradation by ubiquitin-proteasome pathway [31]. Hence, degradation of EZH2 could attenuate breast cancer invasion and metastasis in MCF-7 and MDA-MB-231 cells [32]. Apart from EZH2 degradation mediated by pEZH2-T487 in luminal and TNBC, the biological function of cytoplasm-located pEZH2-T487 in HER2-positive BC progression and metastasis remains unexplored. In our study, we elucidate that cytoplasmic pEZH2-T487 is correlated with HER2 positive status of breast cancer tissues, which might be a potential regulatory target of HER2-positive BC.

As for serine21 of phosphorylated EZH2, views differ in the modulation mechanism between epigenetic and non-epigenetic characteristics. AKT-mediated EZH2 phosphorylation at serine21 drives hepatocarcinogenesis and associates with tumor recurrence and poor survival [33]. In the literature, pS21-EZH2 is functionally important in transactivating Bcl6 and the T follicular helper (T_FH_) cell program, revealing a critical contribution by EZH2 phosphorylation to a non-cancerous, physiological process [34]. Besides, EZH2 activity resulting from posttranslational phosphorylation at the serine-21 site is responsible for the increased enrichment of H3K27me3 at the RECK promoter region, which is related to tumor metastasis and angiogenesis [35]. In a recent study, dephosphorylates EZH2 at S21 could activate its methyltransferase function. Hence, EZH2-S21 phosphorylated-controlled Epithelial–Mesenchymal Transition (EMT) genes are upregulated during fibrotic disease progression of human eyes—3 major extracellular matrix (ECM) target genes: Col1A1, MMP17, and POSTN [36]. However, pEZH2-S21 biological behavior in HER2-positive BCs has not yet been further explored and elucidated. In the present study, we initially suggest that pEZH2-S21, which preferably located in nucleus of breast cancer, is intensively activated in invasive and lymph node metastatic HER2-positive BCs.

It is reported that EZH2-mediated histone modification modulates the response to HER2-targeted therapies via the protein phosphatase 2 A (PP2A) regulatory subunit PPP2R2B regulation [37]. In 2022, researchers demonstrated that inhibition of EZH2 and HDAC could reactivate IFI16-directed immune response of HER2-positive BC patients, changing “cold tumor” into “hot tumor”, and sensitizing anti-HER2 treatment responses [38]. Accordingly, we would speculate that cytoplasic-phosEZH2 and nuclear-phosEZH2 might serve its biological functions separately in invasive BCs, HER2-positive BC particularly.

In conclusion, our study first put forward the clinical significance of phosphorylated EZH2 in HER2-positive BCs. The expression and subcellular localization of phosphorylated EZH2 differs HER2-positive breast cancer invasiveness in a site-specific manner, which associated with metastasis. Further clinical potential therapeutic strategy would be explored in HER2-positive BC to improve the efficacy of target therapy.

## Methods

### Tissue specimen, tissue microarray (TMA) construction and reagents

Breast tumor tissues were collected from our department who underwent breast surgery. In total, 113 patients were recruited from the Second Affiliated Hospital of Zhejiang University between 2020 and 2021. All patients had signed informed consent of the study. Tissue microarray construction was performed with breast benign lesion, paracancerous tissue and breast tumor samples. All these were embedded into paraffin TMA blocks with a manual tissue arrayer. Two TMA cores of 1.0 mm in diameter were sampled from a cohort of 113 breast samples (16 benign tissues, 7 DCIS tissues and 90 breast cancer tissues). Sections of 4 μm were cut and stained with H&E. All the clinical data of these 113 patients has been collected, reviewed and analyzed statistically to testify the clinical importance of sub-location of (p)EZH2 in BC patients, which exhibited in Tables 1 and 2. Three randomly chosen fields were acquired per condition with identical microscope settings and images were scanned measured using Caseviewer software.

### Bioinformatics database analysis

#### Gene expression and survival prognosis analysis

The GEPIA2 database (http://gepia2.cancer-pku.cn) could analysis gene expression profiles from TCGA dataset and Genotype-Tissue Expression (GTEx) projects. EZH2 copy number alteration in pancancers and normal samples from TCGA and GEPIA2 webserver could be obtained by Boxplot [39]. Survival analysis of breast cancer OS, RFS and DMFS with EZH2 expression level were detected by Kaplan-Meier plots. We identified EZH2 expression levels in different subtypes of BC prognosis based on K-M plotter database.

The sequence of phosphorylated EZH2 and schematic diagram is displayed from PhosphoELM and PhosphoSitePlus website database. The association of pEZH2-T487 in breast cancer with clinical features is conducted from CPTAC dataset.

The R package, clusterProfiler, was then used to perform Gene Ontology (GO) and Kyoto Ency- clopedia of Genes and Genomes (KEGG) [40–42] analyses of cancer-related biological processes or pathways affected by EZH2.

### Immunohistochemical (IHC) and immunofluorescence (IF)staining and scoring analysis

They were and paraffin-embedded and sectioned, followed by deparaffinization, rehydration and antigen retrieval. Primary antibodies specific for EZH2, pEZH2-S21, pEZH2-T487 and HER2 were incubated while a negative staining was used as a control. TMA slides were examined and scored by two individual trained pathologists using semi-quantitative IHC and IF scoring system. The IHC score is mainly classified into intensive, moderate and negative based on the staining intensity and fraction of stained cells [43, 44]. Images were acquired with a Zeiss fluorescence microscope. Fields were randomly chosen with the DAPI filter. All screen settings were the same for each condition.

### Statistical analysis

Statistical analysis was performed by SPSS and GraphPad software. To evaluate the significance of phosphorylated EZH2 association with clinicopathological data, Pearson’s Chi-square test and t test were conducted to examine clinical characteristic significance. P < 0.05 was considered as statistically significant.

### Reporting summary

Further information on research design is available in the Nature Research Reporting Summary linked to this article.

### Electronic supplementary material

Below is the link to the electronic supplementary material.

**Supplementary Material 1: Supplementary Fig. 1**. EZH2-related genes in tumors provided by bioinformatics analysis based on TCGA-BRCA using STRING, and metascape database

**Supplementary Material 2: Supplementary Fig. 2**. EZH2 downstream target genes in breast cancer cell examined by RNA sequencing assay and identified using GO enrichment and KEGG pathway, shown as gene correlation network

Supplementary Material 3

## Data Availability

Gene expression data described in this study are deposited in TCGA and GTE database by GEPIA2 (http://gepia2.cancer-pku.cn) website. Survival analysis of EZH2 expression in breast cancer is exported from K-M plotter (https://kmplot.com/analysis/) database. The phosphorylated sites of EZH2 are analyzed from PhosphoSitePlus (http://phosphosite.org) database, PhosphoELM (http://phospho.elm.eu.org) and UALCAN (https://ualcan.path.uab.edu) Clinical Proteomic Tumor Analysis Consortium (CPTAC) database. Additional relevant data that support the findings of this study are available from the corresponding authors upon reasonable request.
