# Correction: Phosphorylation of EZH2 differs HER2-positive breast cancer invasiveness in a site-specific manner

**DOI:** 10.1186/s12885-023-11676-7

**Published:** 2023-12-01

**Authors:** Feng Yu, Lili Li, Mengwen Zhang, Shanshan Sun

**Affiliations:** 1 Department of Colorectal Surgery and Oncology, Key Laboratory of Cancer Prevention and Intervention, The Second Affiliated Hospital, Ministry of Education, Zhejiang University School of Medicine, Hangzhou, Zhejiang, China; Cancer Institute, Zhejiang University, Hangzhou, 310058 Zhejiang China; 2 Department of Medical Oncology, Second Affiliated Hospital, Zhejiang University School of Medicine, Hangzhou, 310058 China; 3 Department of Plastic Surgery, Second Affiliated Hospital, Zhejiang University School of Medicine, Hangzhou, 310058 China; 4 Department of Breast Surgery, Second Affiliated Hospital, Zhejiang University School of Medicine, Hangzhou 310058, China; Key Laboratory of Tumor Microenvironment and Immune Therapy of Zhejiang Province, Second Affiliated Hospital, Zhejiang University School of Medicine, Hangzhou, 310058 China

**Correction: BMC Cancer 23, 948 (2023)**

**https://doi.org/10.1186/s12885-023-11450-9**

Following publication of the original article [1], the authors identified an error the presentation of the author affiliations. The correct affiliations are presented to in this article.

Feng Yu is affiliated to 1, Lili Li is affiliated to 2, Mengwen Zhang is affiliated to 3 and Shanshan Sun is affiliated to 4. The original article [1] has been corrected.
