# Supplementary figures and images for: Phosphorylation of EZH2 differs HER2-positive breast cancer invasiveness in a site-specific manner
Source: BMC Cancer. 2023 Oct 6;23:948. doi: 10.1186/s12885-023-11450-9 (PMC10557267; doi:10.1186/s12885-023-11450-9)

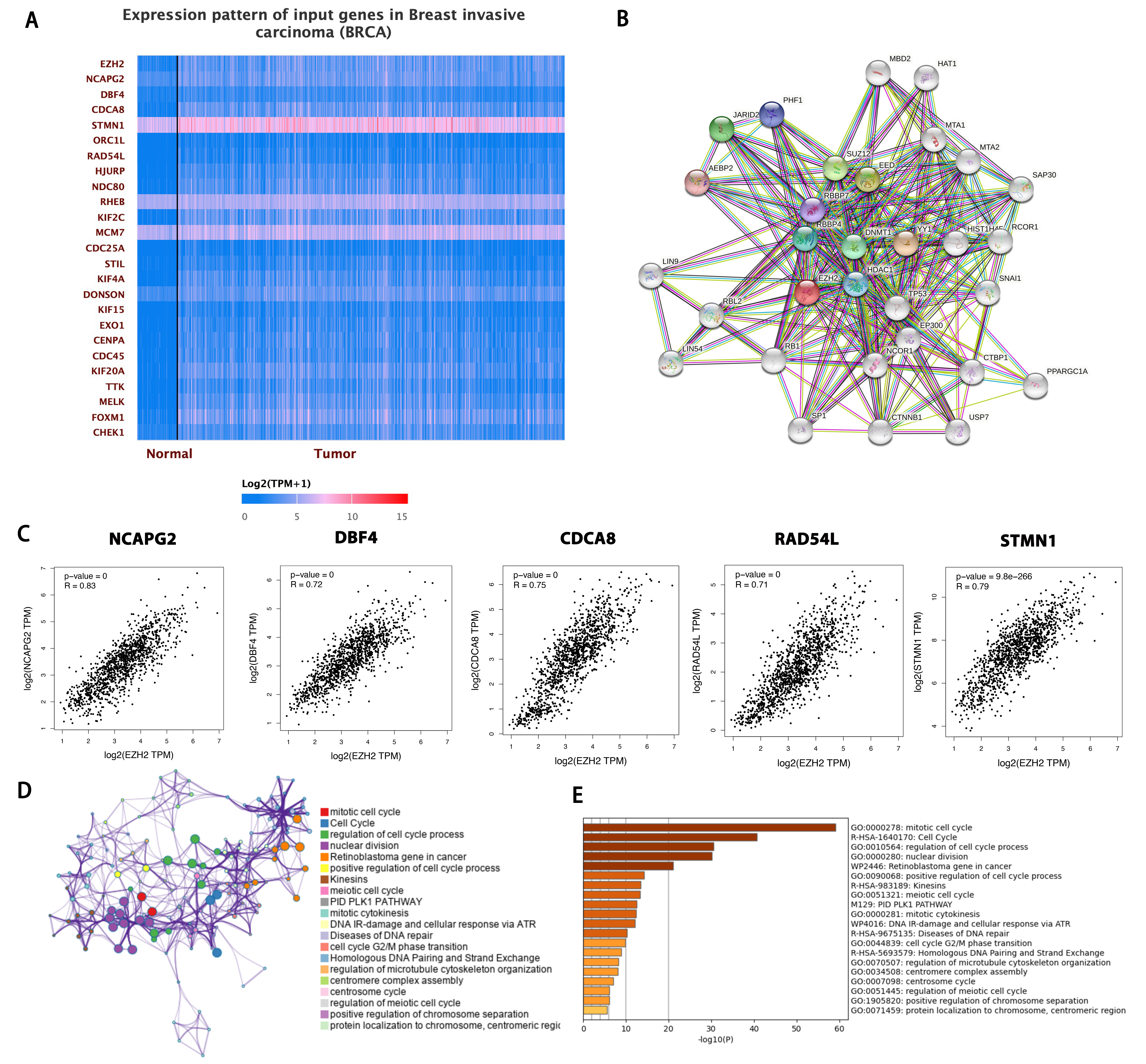

Supplement: Supplementary file 1 — Supplementary Material 1: Supplementary Fig. 1. EZH2-related genes in tumors provided by bioinformatics analysis based on TCGA-BRCA using STRING, and metascape database [file 12885_2023_11450_MOESM1_ESM.jpg]

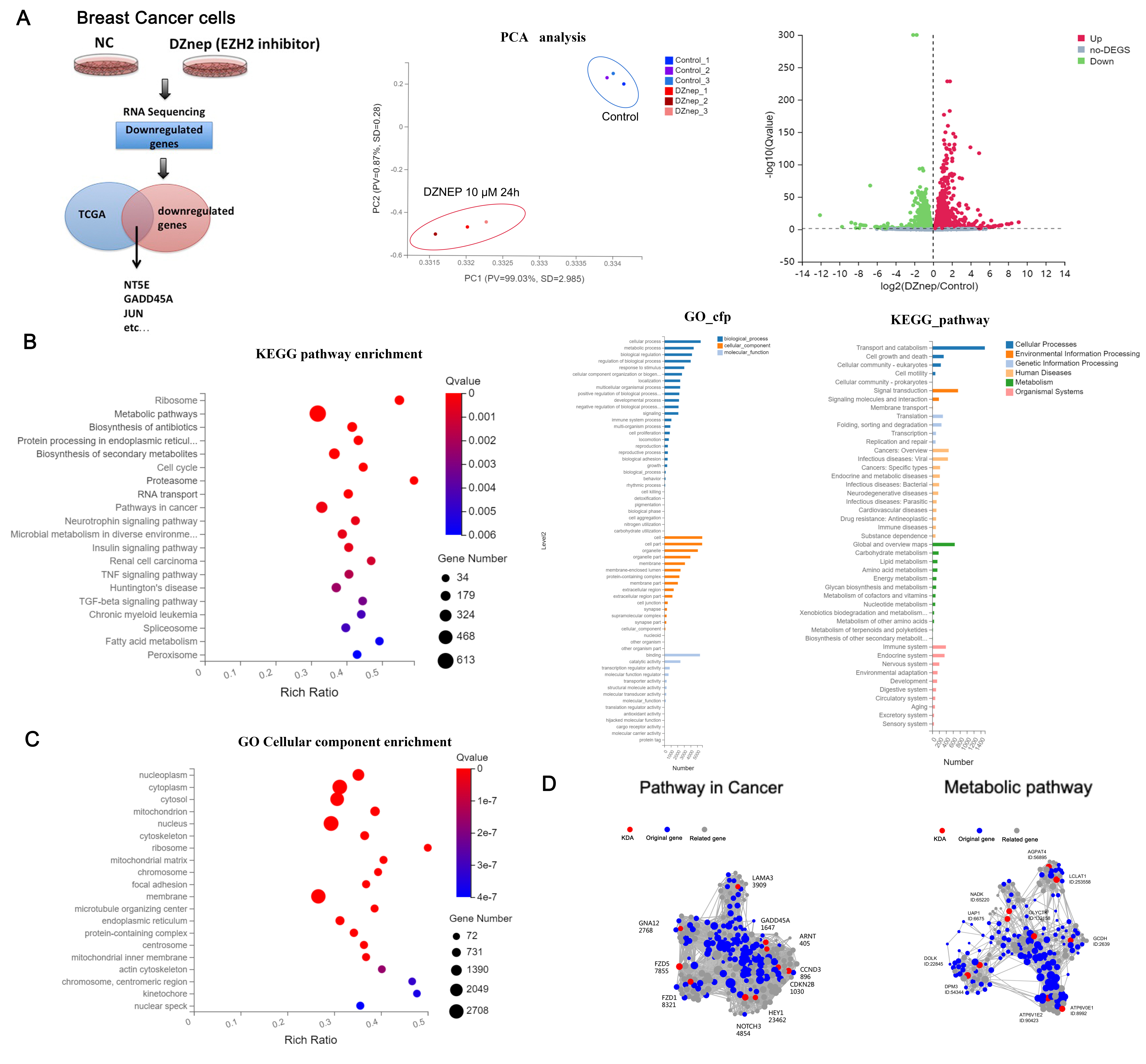

Supplement: Supplementary file 2 — Supplementary Material 2: Supplementary Fig. 2. EZH2 downstream target genes in breast cancer cell examined by RNA sequencing assay and identified using GO enrichment and KEGG pathway, shown as gene correlation network [file 12885_2023_11450_MOESM2_ESM.jpg]
