# Supplementary material for: Phosphorylation of EZH2 differs HER2-positive breast cancer invasiveness in a site-specific manner
Source: BMC Cancer. 2023 Oct 6;23:948. doi: 10.1186/s12885-023-11450-9 (PMC10557267; doi:10.1186/s12885-023-11450-9)
Supplement: Supplementary file 3 — Supplementary Material 3 [file 12885_2023_11450_MOESM3_ESM.docx]

**Supplementary Fig.1**

EZH2-related genes in tumors provided by bioinformatics analysis based on TCGA-BRCA using STRING, and metascape database

**Supplementary Fig.2**

EZH2 downstream target genes in breast cancer cell examined by RNA sequencing assay and identified using GO enrichment and KEGG pathway, shown as gene correlation network.
